# First report of three novel *Bartonella* species isolated in rodents and shrews from nine provinces of Thailand

**DOI:** 10.14202/vetworld.2022.1624-1631

**Published:** 2022-07-08

**Authors:** Decha Pangjai, Burin Nimsuphan, Wimol Petkanchanapong, Wattanapong Wootta, Maskiet Boonyareth, Wuttikon Rodkvamtook, Sumalee Boonmar

**Affiliations:** 1 Department of Medical Sciences, National Institute of Health, Ministry of Public Health, Nonthaburi 11000, Thailand; 2 Department of Parasitology, Faculty of Veterinary Medicine, Kasetsart University, Bangkok 10900, Thailand; 3 Armed Forces Research Institute of Medical Science, Royal Thai Army, Bangkok 10400, Thailand; 4 Akkhraratchakumari Veterinary College, Walailak University, Thasala, Nakhon Si Thammarat 80160, Thailand

**Keywords:** *Bartonella* spp, phylogenetic analysis, polymerase chain reaction, rodents

## Abstract

**Background and Aim:**

*Bartonella* spp. are Gram-negative zoonotic bacteria that are transmitted to humans by several types of animal hosts, including rodents. Several studies have been conducted on the prevalence of *Bartonella* infections in rodents. However, the risk of rodent-associated *Bartonella* spp. infection in humans remains unclear. This study aimed to estimate the prevalence and genetic heterogeneity of *Bartonella* spp. in rodents and shrews from nine provinces of Thailand using culture and molecular techniques.

**Materials and Methods:**

A total of 860 blood samples from rodents and shrews across nine provinces of Thailand were collected from January 2013 to June 2016. *Bartonella* spp. were isolated from all samples using conventional culture techniques and polymerase chain reaction. Phylogenetic tree analysis was used to align the *Bartonella* sequences obtained from this study.

**Results:**

The prevalence of *Bartonella* spp. in rodents and shrews was 11.5% (99/860, 95% confidence interval: 9.38–13.64%). The following nine species of *Bartonella* were detected: *Bartonella tribocorum*, *Bartonella rattimassiliensis*, *Bartonella queenslandensis*, *Bartonella elizabethae*, *Bartonella chanthaburi* spp. nov., *Bartonella satun* spp. nov., *Bartonella coopersplainsensis*, *Bartonella ranong* spp. nov., and *Bartonella henselae*. The prevalence of *Bartonella-*positive animals differed significantly among provinces.

**Conclusion:**

To the best of our knowledge, the three novel *Bartonella* spp. isolated from rodents and shrews across Thailand were detected for the first time in this study. Further studies on the epidemiology of *Bartonella* infection in rodents and its interaction with human health should be conducted in accordance with the Thai government’s “One Health” approach to humans, animals, and the environment.

## Introduction

*Bartonella* spp. are Gram-negative intraerythrocytic bacteria including more than 40 species and subspecies [1]. Several *Bartonella* spp. have been confirmed as zoonotic pathogens, such as *Bartonella elizabethae*, *Bartonella tribocorum*, *Bartonella* *henselae*, *Bartonella* *vinsonii*. Sub spp. *arupensis*, and *Bartonella* *tamiae*, most of which are transmitted by reservoir hosts and blood-sucking arthropods [2].

Rodents are known to be the main reservoir hosts for different *Bartonella* spp.; however, some species involve other animals as well. *B. henselae* utilizes cats and *Bartonella* *bovis* and *Bartonella* *chomelii* utilize cattle as reservoirs [3]. Several *Bartonella* spp. have been isolated from rodents in several countries, including Thailand [4–9]. These pathogens are associated with various human diseases, such as cat scratch disease (*B. henselae*), trench fever (*Bartonella quintana*), Oroya fever (*Bartonella bacilliformis*), and endocarditis (*B. tamiae*) [10–12]. In particular, past exposure to rats has been reported in three patients from Thailand with fever, myalgia, and headache [13]. Several reports of these infections in rodents in Thailand have been described [6–9, 12–15]. However, the risk of rodent-associated *Bartonella* spp. infection in humans remains unclear.

The study aimed to estimate the prevalence and genetic heterogeneity of *Bartonella* spp. in rodents and shrews from nine provinces of Thailand using culture and molecular techniques and phylogenetic analysis.

## Materials and Methods

### Ethical approval

The study was approved by the Institutional Animal Care and Use Committee of the National Institute of Health (NIH), Thailand.

### Study period and location

**Figure-1:**
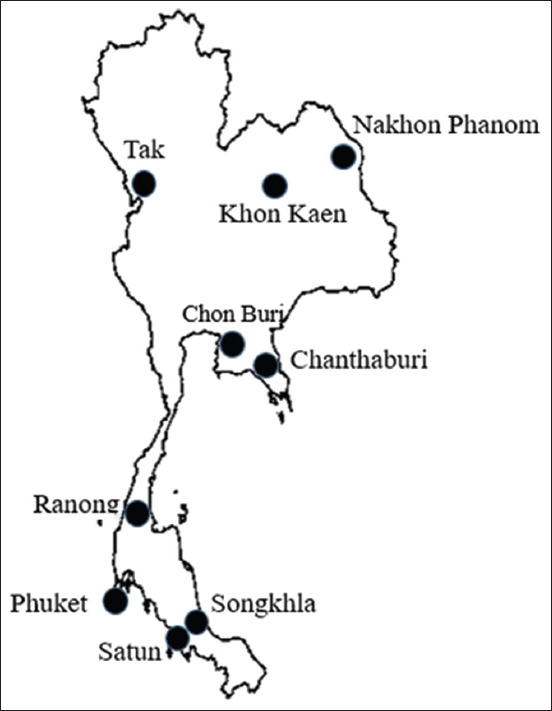
Geographic locations of nine provinces in Thailand where rodents and shrews were captured for this study [Source: http://geosurin.blogspot.com/2009/09/blog-post-17.html].

The study was conducted from January 2013 to June 2016. The blood samples were collected from nine provinces of Thailand; Khon Kaen, Nakhon Phanom, Tak, Chon Buri, Chanthaburi, Ranong, Phuket, Songkhla, and Satun (Figure-1). The samples were processed at the Department of Medical Sciences, NIH Laboratory, Ministry of Public Health, Nonthaburi, Thailand.

### Sample collection

We calculated the minimum sample size based on a previous study by Pangjai *et al*. [6] using the Epitools program (www.epitool.net) with 95% confidence interval (CI) and 2.5% precision. Based on the results, 401 samples should have been collected. Overall, 860 small mammals, comprising 800 rodents and 60 shrews, were captured using traps from nine provinces of Thailand. Animal species were identified by their morphological characteristics before they were euthanized using a Carbon oxide (CO_2_) chamber. A total of 0.5–2 mL of blood samples were aseptically collected through cardiopuncture and immediately placed in sterile ethylenediamine tetra-acetic acid tubes. The samples were transported to the Department of Medical Sciences, NIH Laboratory under chilled conditions and stored at −20°C until further processing.

### Isolation of *Bartonella*

*Bartonella* was isolated according to a previously described method [16] with slight modifications. Briefly, frozen blood samples were thawed at 25°C, and 200 mL of each sample was centrifuged at 1,800x*g* for 70 min. The sediment was mixed with an equal volume of Medium 199 (Life Technologies, USA) supplemented with sodium pyruvate and fetal bovine serum (Life Technologies, United States). The mixture was then inoculated onto brain heart infusion agar (BHIA, Difco, United States) plates containing 5% defibrinated rabbit blood. The plates were incubated at 35°C under 5% CO_2_ for 2–4 weeks. Consequently, Gram-negative coccobacilli grew as small, rough, and grayish colonies and required long culture periods, which were tentatively considered as *Bartonella* species. The bacteria were subcultured in fresh media and all isolates were maintained in Trypticase Soy Broth with 20% glycerol (v/v) for further characterization.

### DNA extraction and polymerase chain reaction (PCR) amplification

The genomic *Bartonella* DNA was detected using specific PCR primers as described previously by Boonmar *et al*. [16]. Genomic DNA was extracted from each isolate using InstaGene Matrix (BioRad, Hercules, United States). Primers targeting the β-subunit of RNA polymerase (*rpoB*) [17] (primer pair sequences, 5´-CGCATTGGCTTACTTCGTATG-3´ and 5´-GTAGACTBATTAGAACGCTG-3´) and citrate synthase (*gltA*) [18] (primer pair sequences, 5´-AATGCAAAAAGAACAGTAAACA-3´ and 5´-GGGGACCAGCTCATGGTGG-3´) were used for PCR. PCR was performed using 20 μL of reaction mixtures containing 20 ng of extracted DNA, 200 μmol/L of each deoxynucleotide triphosphate, 1.5 mmol/L of MgCl_2_, 0.5 U of Go-Taq DNA polymerase (Promega, Madison, Wisconsin, United States), and 1 pmoL of each primer. The thermal cycling conditions of PCR included a denaturation step at 94°C for 2 min, followed by 35 cycles of 94°C for 30 s, 53°C for 30 s, and 72°C for 1 min, with a final step of 72°C for 7 min. Positive and negative controls were included in each experiment. Finally, 10 μL of each PCR product was subjected to electrophoresis on 1.5% agarose gels containing ethidium bromide and visualized on an ultraviolet transilluminator. The expected length of PCR products was 825 bp (*rpoB* primers) and 379 bp (*gltA* primers).

### Phylogenetic analysis

The Clustal X program [19] was used to align *Bartonella* sequences obtained from this study. The data will be deposited in the GenBank/EMBL/DDBJ databases. A phylogenetic tree was drawn based on the aligned sequences of *gltA* and *rpoB* genes using the neighbor-joining method with Kimura’s two-parameter distance method in MEGA 11 [20]. Bootstrap analysis was conducted using 1,000 resamples. The *Brucella melitensis* strain 16M sequence was used as an out-group.

### Statistical analysis

Pearson’s Chi-square test and Fisher’s exact test were used to comparatively analyze the prevalence of animal species among provinces using the IBM SPSS Statistics software. The differences observed were considered statistically significant at p ≤ 0.05.

## Results

A total of 860 small mammals were captured from nine provinces of Thailand, including 399 *Rattus* spp., 50 *Bandicota* spp., 351 other spp., and 60 *Suncus murinus* (shrews). Overall, 11.5% of blood samples from rodents (99/860, 95% CI: 9.38–13.64%) were positive for nine *Bartonell*a species; the rodents included 86/399 *Rattus* spp. (21.5%), 5/50 *Bandicot*a spp. (10%), 3/29*9 Mus musculu*s (1.0%), and 5/60 *Suncus murinus* (8.3%). The incidence and identities of the nine *Bartonella* spp. were as follows: 27.3% of *B. tribocorum*, 20.2% of *Bartonella rattimassiliensis*, 15.2% of *Bartonella queenslandensis*, 10.1% of *B. elizabethae*, 8.1% of *Bartonella chanthaburi* spp. nov., 6.1% of *Bartonella satun* spp. nov., 6.1% of *Bartonella coopersplainsensis*, 5.1% of *Bartonella ranong* spp. nov., and 2.0% of *B. henselae* (Table-1).

**Table 1:** Prevalence of nine *Bartonella* species from 860 rodents and shrews of nine provinces in Thailand.

Host species	Number of examined	Number of positive (%)	Number of animals infected with *Bartonella* species								

			*Bartonella cooperspl* *ainsensis*	*Bartonella elizabethae*	*Bartonella henselae*	*Bartonella queensla ndensis*	*Bartonella rattimassi liensis*	*Bartonella tribocorum*	*Bartonella chanthaburi* spp. nov.	*Bartonella satun* spp. nov.	*Bartonella ranong* spp. nov.
*Bandicota indica*	46	5 (11.1)	1	0	0	2	1	1	0	0	0
*Bandicota savilei*	4	0	0	0	0	0	0	0	0	0	0
*Berylmys berdmorei*	5	0	0	0	0	0	0	0	0	0	0
*Callosciurus notatus*	3	0	0	0	0	0	0	0	0	0	0
*Crocidura fuliginosa*	23	0	0	0	0	0	0	0	0	0	0
*Maxomys rajah*	5	0	0	0	0	0	0	0	0	0	0
*Maxomys surifer*	1	0	0	0	0	0	0	0	0	0	0
*Menetes berdmorei*	3	0	0	0	0	0	0	0	0	0	0
*Mus caroli*	10	0	0	0	0	0	0	0	0	0	0
*Mus musculus*	299	3 (1.0)	0	0	0	0	0	3	0	0	0
*Rattus argentiventer*	5	0	0	0	0	0	0	0	0	0	0
*Rattus exulans*	125	8 (6.4)	0	0	0	0	0	8	0	0	0
*Rattus norvegicus*	59	28 (47.5)	0	8	1	8	2	9	0	0	0
*Rattus rattus*	171	48 (28.1)	5	2	1	5	17	6	7	5	0
*Rattus tenezumi*	39	2 (5.1)	0	0	0	0	0	0	1	1	0
*Tupaia glis*	2	0	0	0	0	0	0	0	0	0	0
*Suncus murinus*	60	5 (8.3)	0	0	0	0	0	0	0	0	5
Total	860	99 (11.5)	6 (6.1)	10 (10.1)	2 (2.0)	15 (15.2)	20 (20.2)	27 (27.3)	8 (8.1)	6 (6.1)	5 (5.1)

**Figure-2:**
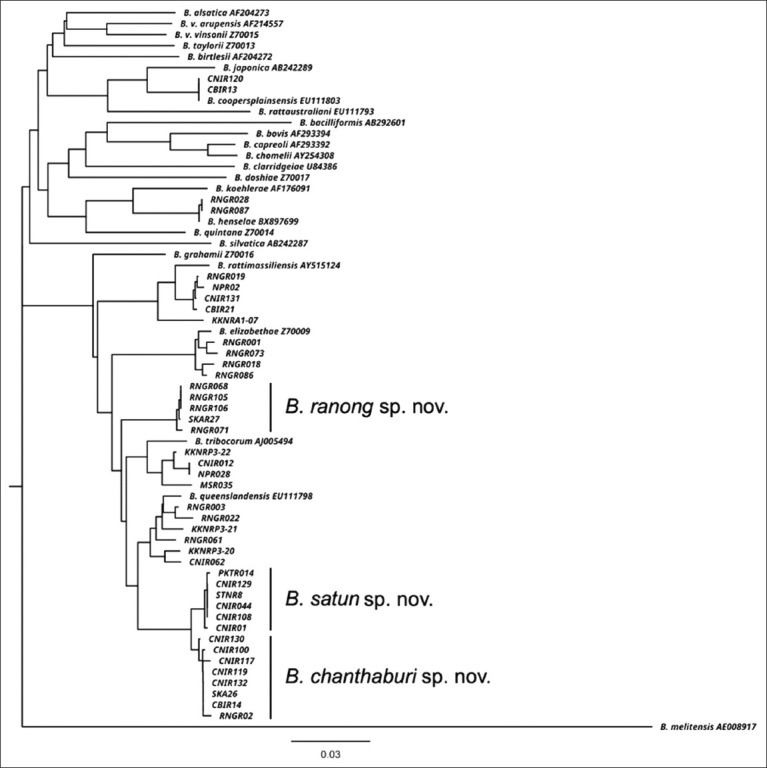
Neighbor-Joining tree based on the concatenated sequences of 2 loci (*gltA* and *rpoB*) of *Bartonella* species. The phylogenetic tree was reconstructed using the Kimura 2-parameter substitution model. The tree was rooted using *Brucella melitensis* as an out-group. Bootstrap values resulting from 1000 bootstrap trials are indicated for each branch. Bar represents 0.03 estimated nucleotide substitutions per site. gltA=Citrate synthase, rpoB=RNA polymerase.

Table-2 shows the geographic distribution of the nine *Bartonella* spp. isolated from rodents and shrews. Of all animals carrying these pathogens, 22/414 (5.31%) were captured in the northeastern region of Thailand, 1/41 (2.5%) in the northern region, 5/40 (12.5%) in the central region, 25/148 (16.9%) in the eastern region, and 46/217 (21.2%) in the southern region. The prevalence of the nine *Bartonella* spp. in the nine provinces was as follows (in descending order): 35.1% (40/114 animals) in Ranong, 31.0% (14/45) in Nakhon Phanom, 16.9% (25/148) in Chanthaburi, 12.5% (5/40) in Chonburi, 7.7% (3/39) in Phuket, 5.4% (2/37) in Songkhla, 3.7% (1/27) in Satun, 2.5% (1/41) in Tak, and 2.2% (8/369) in Khon Kaen. Among the northeastern provinces, *Bartonella* prevalence in Nakhon Phanom was significantly higher than that in Khon Kaen (p < 0.001). Further, among the southern provinces, the prevalence in Ranong was significantly higher than that in Satun (p < 0.001). The phylogenetic tree of the 99 *Bartonella*-positive sequences of gltA and rpoB fragments is shown in Figure-2. Table-3 shows the GenBank accession numbers of the nucleotide sequences obtained from this study, which were deposited in the GenBank.

**Table 2:** Geographic distribution of nine *Bartonella* species isolated from rodents and shrews in nine provinces, Thailand.

	Province	Number examined	Number of positive (%)	Number of animals infected with Bartonella species								
		
				*Bartonella coopersplain sensis*	*Bartonella elizabethae*	*Bartonella henselae*	*Bartonella queenslan densis*	*Bartonella rattimassi liensis*	*Bartonella tribocorum*	*Bartonella chanthaburi* spp. nov.	*Bartonella satun spp*. nov	*Bartonella ranong* spp. nov
North- Eastern	Khon Kaen	369	8 (2.2)*	0	0	0	3/(Rr = 1, Bi = 2)	2/(Bi = 1, Rr = 1)	3/(Mm = 3)	0	0	0
	Nakhon Phanom	45	14 (31)*	0	0	0	0	3/(Rr = 3)	11/(Re = 8, Rn = 1, Rr = 2)	0	0	0
	subtotal	414	22 (5.31)	0	0	0	3/(Rr = 1, Bi = 2)	5(Bi = 1, Rr = 4)	14 (Mn = 3, Re = 8, Rn = 1, Rr = 2)	0	0	0
Northern Central Eestern	Tak	41	1 (2.5)	0	0	0	0	0	1/(Bi = 1)	0	0	0
	Chon Buri	40	5 (12.5)	2/(Bi = 1, Rr = 1)	0	0	0	2/(Rr = 2)	0	1/(Rr = 1)	0	0
	Chanthaburi	148	25 (16.9)	4/(Rr = 4)	0	0	2/(Rr = 2)	8/(Rr = 8)	2/(Rr = 2)	5/(Rr = 5)	4/(Rr = 4)	0
Southern	Ranong	114	40 (35.1)**	0	10/(Rn = 8, Rr = 2)	2/(Rn = 1, Rr = 1)	10/(Rn = 8, Rr = 2)	3/(Rn = 2, Rr = 1)	10/(Rn = 8, Rr = 2)	1/(Rr = 1)	0	4/(Sm = 4)
	Phuket	39	3 (7.7)	0	0	0	0	2/(Rr = 2)	0	0	1/(Rr = 1)	0
	Songkhla	37	2 (5.4)	0	0	0	0	0	0	1/(Rt = 1)	0	1/(Sm = 1)
	Satun	27	1 (3.7)**	0	0	0	0	0	0	0	1/(Rt = 1)	0
	Subtotal	217	46 (21.2)	0	10/(Rn = 8, Rr = 2)	2/(Rn = 1, Rr = 1)	10/(Rn = 8, Rr = 2)	5/(Rn = 2, Rr = 3)	10/(Rn = 8, Rr = 2)	2/(Rr = 1, Rt = 1)	2/(Rr = 1, Rt = 1)	5(Sm = 5)
	Total	860	99 (11.5)	6 (6.1)	10 (10.1)	2 (2.0)	15 (15.2)	20 (20.2)	27 (27.3)	8 (8.1)	6 (6.1)	5 (5.1)

* Prevalence in NakhonPhanom was significantly higher than in KhonKaen (p < 0.001),

** Prevalence in Ranong was significantly higher than in Satun (p < 0.001)

**Table 3:** GenBank accession numbers for nucleotide sequences.

Nucleotide sequences	GenBank accession numbers	

	gltA	rpoB
*B. elizabethae_THRNGR001*	MF105784	MF105860
*B. queenslandensis_THRNGR003*	MF105785	MF105861
*B. tribocorum_THRNGR006*	MF105786	MF105862
*B. tribocorum_THRNGR012*	MF105787	MF105863
*B. elizabethae_THRNGR018*	MF105788	MF105864
*B. rattimassiliensis_THRNGR019*	MF105789	MF105865
*B. tribocorum_THRNGR020*	MF105790	MF105866
*B. queenslandensis_THRNGR022*	MF105791	MF105867
*B. henselae_THRNGR028*	MF105792	MF105869
*B. elizabethae_THRNGR029*	MF105793	MF105870
*B. rattimassiliensis_THRNGR032*	MF105794	MF105871
*B. tribocorum_THRNGR033*	MF105795	MF105872
*B. queenslandensis_THRNGR034*	MF105796	MF105873
*B. elizabethae_THRNGR036*	MF105797	MF105874
*B. queenslandensis_THRNGR037*	MF105798	MF105875
*B. elizabethae_THRNGR043*	MF105799	MF105876
*B. tribocorum_THRNGR044*	MF105800	MF105877
*B. elizabethae_THRNGR045*	MF105801	MF105937
*B. queenslandensis_THRNGR061*	MF105802	MF105878
*B. ranong_THRNGR068*	MF105803	MF105879
*B. ranong_THRNGR071*	MF105804	MF105880
*B. elizabethae_THRNGR073*	MF105805	MF105881
*B. queenslandensis_THRNGR074*	MF105806	MF105882
*B. tribocorum_THRNGR077*	MF105807	MF105884
*B. tribocorum_THRNGR079*	MF105808	MF105885
*B. tribocorum_THRNGR080*	MF105809	MF105886
*B. queenslandensis_THRNGR081*	MF105810	MF105887
*B. tribocorum_THRNGR083*	MF105811	MF105888
*B. elizabethae_THRNGR084*	MF105812	MF105889
*B. elizabethae_THRNGR086*	MF105813	MF105890
*B. queenslandensis_THRNGR091*	MF105814	MF105892
*B. tribocorum_THRNGR094*	MF105815	MF105893
*B. ranong_THRNGR105rpoB*	MF105816	MF105894
*B. ranong_THRNGR106rpoB*	MF105817	MF105895
*B. rattimassiliensis_THPKTR006*	MF105818	MF105896
*B. satun_THPKTR014*	MF105819	MF105897
*B. rattimassiliensis_THCTIR99*	MF105820	MF105898
*B. chanthaburi_THCTIR100*	MF105821	MF105899
*B. coopersplainsensis_THCTIR101*	MF105822	MF105900
*B. rattimassiliensis_THCTIR103*	MF105823	MF105901
*B. rattimassiliensis_THCTIR105*	MF105824	MF105902
*B. rattimassiliensis_THCTIR106*	MF105825	MF105903
*B. rattimassiliensis_THCTIR107*	MF105826	MF105904
*B. satun_THCTIR108rpoB*	MF105827	MF105905
*B. chanthaburi_THCTIR119*	MF105829	MF105906
*B. coopersplainsensis_THCTIR120*	MF105830	MF105907
*B. coopersplainsensis_THCTIR128*	MF105831	MF105908
*B. chanthaburi_THCTIR129*	MF105832	MF105909
*B. chanthaburi_THCTIR130*	MF105833	MF105910
*B. rattimassiliensis_THCTIR131*	MF105834	MF105911
*B. chanthaburi_THCTIR132*	MF105835	MF105912
*B. rattimassiliensis_THCTIR135*	MF105836	MF105913
*B. rattimassiliensis_THCTIR141*	MF105837	MF105914
*B. queenslandensis_THKKNRP3-20*	MF105838	MF105915
*B. queenslandensis_THKKNRP3-21*	MF105839	MF105916
*B. tribocorum_THKKNRP3-22*	MF105840	MF105917
*B. tribocorum_THKKNRP3-24*	MF105841	MF105918
*B. tribocorum_THKKNRP3-25*	MF105842	MF105919
*B. queenslandensis_THKKNRP3-29*	MF105843	MF105920
*B. satun_THSTNR8*	MF105844	MF105921
*B. chanthaburi_THSKAR26*	MF105845	MF105922
*B. ranong_THSKAR27*	MF105846	MF105923
*B. tribocorum_THMSR035*	MF105847	MF105924
*B. satun_THCTIR01*	MF105848	MF105925
*B. tribocorum_THCTIR012*	MF105849	MF105926
*B. tribocorum_THCTIR043*	MF105850	MF105927
*B. satun_THCTIR044*	MF105851	MF105928
*B. queenslandensis_THCTIR062*	MF105852	MF105929
*B. queenslandensis_THCTIR064*	MF105853	MF105930
*B. coopersplainsensis_THCBIR13*	MF105854	MF105931
*B. chanthaburi_THCBIR14*	MF105855	MF105932
*B. coopersplainsensi_sTHCBIR20*	MF105856	MF105933
*B. rattimassiliensis_THCBIR21*	MF105857	MF105934
*B. rattimassiliensis_THCBIR23*	MF105858	MF105935
*B. rattimassiliensis_THKKNRA1-07*	MF105859	MF105936

gltA=Citrate synthase, rpoB=RNA polymerase, *B. tribocorum=Bartonella tribocorum, B. rattimassiliensis=Bartonella rattimassiliensis, B. queenslandensis=Bartonella queenslandensis, B. elizabethae=Bartonella elizabethae, B. chanthaburi=Bartonella chanthaburi, B. satun=Bartonella satun, B. coopersplainsensis=Bartonella coopersplainsensis, B.ranong=Bartonella ranong, B. henselae=Bartonella henselae*

## Discussion

The prevalence of rodent-associated *Bartonella* spp. has shown high diversity, with more than 20 such species reported worldwide. It is known that more than one *Bartonella* spp. can circulate in rodent communities, and the presence of multiple *Bartonella* genotypes in the same host has been reported [3, 5, 21], leading to emerging bartonellosis, particularly in Southeast Asia [22, 23]. The prevalence of these pathogens was reported to be 6% in Indonesia [24], 9.3–42.9% in China [25], 10.1–30.4% in Lao PDR [26], and 13.5–13.8% in Malaysia [27], depending on the diagnostic method, location, environmental conditions, presence of vectors, and animal host species and their habitats. The prevalence of 11.5% reported in this study is similar to that reported in a study conducted in Malaysia [27]. In line with the previous studies, *Bartonell*a spp. in our study was also frequently isolated from *Rattus rattu*s [7, 8]; however, other studies showed contrasting results [24–26]. The previous studies from Thailand have identified the presence of *B. elizabethae*, *B. henselae*, *Bartonella* *clarridgeae*, and *B. tamiae*, which were known to cause infections in humans [6–9, 12–13]. Of these, we did not detect *B. tamiae* and *B. clarridgeae* in this study but we found the other two species in addition to *B. tribocorum*, *B. rattimassiliensis*, *B. coopersplainsensis*, *B. queenslandensis*, and three novel *Bartonella* spp.

*B. henselae* is a well-known pathogen in wild and domestic cats and causes cat-scratch disease [28, 29]. It has been isolated from rodents in Thailand in a previous study [7, 8] and is associated with febrile Thai patients [13, 30]. This pathogen was detected in approximately 2% of the rodents from the Ranong Province. Notably, the three novels *Bartonella* spp. were also found in this province and were isolated from shrews.

*B. elizabethae* is widely distributed in Asian countries [7, 8, 25]. It is known to be associated with endocarditis [31] and human neuroretinitis [32]. We found this species in approximately 10% of the rodents in the Ranong province near the Myanmar border, where there are several markets, which are visited by business travelers and workers. The Ranong Province showed the highest prevalence of *Bartonella* infection in animals (35.1%) among all provinces. Thus, the epidemiology of this infection in febrile patients with rodent exposure should be considered.

In this study, *B. tribocorum* was the most prevalent *Bartonella* spp. in rodents (27.3%) followed by *B. rattimassiliensis* (20.2%), both of which had been detected in febrile patients in Thailand in a previous study [13]. Almost all incidences of these species were in *Rattus* rodents, similar to that reported in the previous studies [4, 26, 30, 33].

The other two *Bartonella* spp.; *B. queenslandensis* and *B. coopersplainsensis* were also isolated from *Rattus* rodents. They have been isolated from rodents and fleas in Taiwan in a previous study [34].

We found three novel *Bartonella* spp. in the Chanthaburi, Satun, and Ranong provinces. The public health information concerning *Bartonella* infections in these three provinces remains unknown. Further collaboration between human and animal sectors can help investigate the possibility of new *Bartonella* spp. infections in febrile patients with rodent exposure in these three provinces.

## Conclusion

To the best of our knowledge, this is the first study that reported the detection of three novel *Bartonella* spp. isolated from rodents and shrews in Thailand. In this study, nine different *Bartonella* spp. were detected and most of them were potentially zoonotic, using rodents as reservoir hosts. Further studies on the risk of this infection among humans, rodents, and the environment are needed to advance public health information.

## Authors’ Contributions

DP, BN, WP, WW, and NC: Collected the samples. DP and WR: Provided technical help during the experiments. PW and MB: Did the statistical analysis. SB: Designed the study and drafted and revised the manuscript. All authors have read and approved the final manuscript.
